# Prevalence and socio-ecological correlates of adolescent alcohol, tobacco, and marijuana use in the Turks and Caicos Islands

**DOI:** 10.3389/fpubh.2026.1747234

**Published:** 2026-05-29

**Authors:** Shandey D. Malcolm, Jennifer L. Brown, Alicia B. Malcolm, Sasha Walrond, Kendra O. Malcolm, Kayan S. Garland, Natasha Robinson, Monica L. Kasting, Yumary Ruiz, Donald T. Simeon

**Affiliations:** 1 Department of Public Health, Purdue University, West Lafayette, IN, United States; 2 Department of Psychological Sciences, Purdue University, West Lafayette, IN, United States; 3 Ministry of Health and Human Services, Grand Turk, Turks and Caicos Islands; 4 Caribbean Centre for Health Systems Research and Development, University of West Indies, St. Augustine, Trinidad and Tobago

**Keywords:** adolescent risk behaviors, global school-based health survey, socioecological model, substance use, Turks and Caicos Islands

## Abstract

**Introduction:**

Adolescent substance use is high in the Caribbean. To inform policy and programming, it is necessary to understand these behaviors at the country level. However, little is known about adolescent substance use in small island states. For the first time, we determined the prevalence and correlates of adolescent substance use in the Turks and Caicos Islands (TCI).

**Methods:**

We used secondary data from the first Global School-based Health Survey, conducted from May to June 2022. All students in all schools were invited to complete the cross-sectional survey in their classrooms. The student response rate was 66.5%; *N* = 1,684, M_age_ = 14.3 years, 49.82% male. We determined the frequency of recent alcohol, tobacco, and marijuana use, defined as any use in the previous 30 days (yes/no). Informed by the socioecological model, we considered individual factors (sex, age, food scarcity, bullying victimization, and psychological distress) and interpersonal factors (peer support and parent-adolescent relationship quality) as predictors of substance use in logistic regression models.

**Results:**

Recent alcohol, tobacco, and marijuana use was reported by 29.11, 12.05, and 9.01% of adolescents, respectively. Older age, psychological distress, and lower parental involvement were associated with alcohol use. Psychological distress and lower parental involvement were associated with tobacco use. Finally, male sex, older age, psychological distress, bullying victimization, and lower parental involvement were associated with marijuana use.

**Conclusions:**

Our study demonstrates substance use above global averages and suggests interventions primarily focused on promoting parental involvement and preventing psychological distress. Further qualitative and quantitative interviews will provide more informed intervention strategies.

## 1 Introduction

Approximately 106 million adolescents, defined by the World Health Organization (WHO) as persons 10–19 years old, live in Latin America and the Caribbean (LAC) (1, 2). This population reports high rates of health risk behaviors, including substance use. The Global School-based Health Survey (GSHS), developed by the WHO, is a standardized tool used to collect information on health behaviors among adolescents worldwide, and thus allows for global and regional comparisons (3). Using this data, the 2006–2013 pooled estimate for drinking alcohol at least once in the past 30 days (34.9%) and ever being drunk (22.2%) among adolescents 12–15 years was found to be higher in LAC than all WHO regions combined (25% and 17.5%, respectively) (4). Tobacco use among adolescents aged 13–15 years between 2000 and 2025 was also found to be higher in LAC (11.3%) than global averages (10.3%) (5). Finally, when examining 5 of the 8 WHO regions between 2012 and 2022, Wang and Wang (6) found that the median prevalence of recent 30-day marijuana use among adolescents (12–17 years) was higher in the LAC (5.1%) than in all regions combined (4.3%). Thus, LAC reports higher rates of the three most used substances among adolescents: alcohol, marijuana, and tobacco (7).

Regional reports on the prevalence of substance use behaviors disproportionately represent larger countries in LAC. Smaller territories often lack national data on adolescent health behaviors because of limited human and financial resources (8). For instance, the Turks and Caicos Islands (TCI), a British Overseas Territory in the Caribbean with an estimated 2022 population of 47, 720, had never conducted the GSHS (9). Adolescent substance use rates in this country are likely closer to other LAC countries than global averages, given a similar history and a shared geographical location. It is also likely that variations exist due to differences in national policies and cultural norms. Indeed, Ma et al. (4) found that the 30-day alcohol consumption among 13–15-year adolescents ranged from 16% in Guatemala to 52% in St. Lucia and Jamaica between 2006 and 2013. Similarly, Wang and Wang (6) found that tobacco use in LAC ranged from 3.9% in Suriname to 14% in Jamaica and that lifetime marijuana use ranged from 11.9% in the Dominican Republic to 19.4% in Jamaica. These data demonstrate that among LAC, rates are highest in the English-speaking Caribbean (ESC), but that there is a scarcity of substance use prevalence data in smaller ESC territories like the TCI. Given the variation, they also highlight the need to understand prevalence rates at the country's level. Accurate epidemiological data on adolescent substance use are vital for informing public health practice, including the identification of priorities for policy action and guiding the design of prevention and intervention programs. Our focus on substance use also aligns with the United Nations Sustainable Development Goal 3 (target 3.5), to strengthen the prevention and treatment of substance abuse (10).

Understanding adolescent substance use prevalence rates at the national level is especially critical, given the associated adverse effects. Adolescent substance use is associated with mental health disorders such as depression and anxiety, physical consequences such as road traffic accidents and sexually transmitted diseases, and social outcomes such as lower educational attainment and criminal activity (11–13). Moreover, adolescent substance use can lead to substance use disorders throughout the life course (14). In the ESC, the high rate of risk behaviors, including substance use, has translated to poor health outcomes. This region has the second-highest rate of adolescent mortality in the world, with the leading causes of death all commonly linked to substance use: injury, violence, drowning, and suicide (15). In the TCI, there has been a steady increase in adolescent mortality primarily due to homicides and violent crimes (16). Targeted and informed interventions will depend on understanding the specific factors and circumstances that increase risk.

The socio-ecological model (SEM) provides a valuable framework for understanding factors at various levels within one's environment that may be associated with differential patterns of adolescent substance use (17). It recognizes that multiple factors, including individual characteristics (such as age, sex, socioeconomic status, bullying victimization, and psychological distress) and interpersonal factors (such as peer and parental relationships), influence adolescent health behaviors such as substance use. Among the limited studies available on adolescent substance use in the ESC, it is evident that individual-level factors differ at the country level. For instance, older age is associated with recent 30-day marijuana use in Jamaica but not in Barbados, and higher rates of marijuana use among males exist in some but not all countries (18–20). Likewise, bully victimization was associated with marijuana use in Saint Vincent and the Grenadines but not in Jamaica (21, 22). The influence of interpersonal factors on substance use behaviors also varies across the region. Ruprah et al. (23) found that engaged parenting protects against problematic alcohol consumption in some (Barbados, Grenada, Jamaica, and Trinidad and Tobago) but not all (Antigua and Suriname) LAC countries. Finally, peer factors increased the odds of marijuana use in Barbados but were protective in Jamaica (18, 20).

The few studies examining correlates of adolescent substance use in the ESC underscore the relevance of the local context. They also represent a few relatively large regional countries (e.g., populations greater than 100,000). There is a limited focus on correlates in smaller territories such as the TCI, where adolescent outcomes are also worsening, and targeted prevention strategies are needed. The highlighted studies also feature data collection well before the COVID-19 pandemic and the associated stay-at-home orders. Emerging research from across the globe illustrates that COVID-19 is linked to poor outcomes among adolescents, including substance use (24). More recent studies are needed to better understand adolescent health in the post-COVID-19 era. Our study aims to understand the prevalence and correlates of alcohol, tobacco, and marijuana use in TCI adolescents following COVID-19. While we anticipate some variation due to differences in cultural norms and values, we hypothesize that the substance use rates will be closer to regional averages in LAC and ESC than global averages. Although some differences may emerge regarding alcohol, tobacco, and marijuana use, given differences in social acceptability and risk perceptions (25, 26), we further hypothesize that substance use will be related to older age, male sex, low socioeconomic status, bullying victimization, psychological distress, poor parental relationships, and a limited number of friends.

## 2 Materials and methods

### 2.1 Study participants

We utilized secondary data from the first Turks and Caicos Islands (TCI) Global School-Based Health Survey (GSHS). The TCI GSHS was administered for the first time between May 18th and June 10th, 2022, in accordance with standardized procedures developed by the WHO (3). A census approach was utilized rather than the traditional two-stage random sample design, given the relatively small size of the high school population (about 2,500 students). There are 17 high schools throughout TCI. Since one high school was too small to ensure confidentiality, it was excluded, leaving 16 schools invited to participate in the study. The school response rate was 81.2% (i.e., 13 of 16 schools agreed). The three schools that declined participation represented a small proportion (3.7%) of the TCI High school population. Parents received information about the survey through participating schools. They were also sent a copy of the instrument and a form, which they completed and signed to opt their child out of participation. During survey administration, students were informed of the survey's purpose and of the measures in place to protect their confidentiality. Specifically, no identifying information would be collected, and completed forms would be placed in a receptacle rather than collected by survey administrators. They also reminded students that participation was voluntary and that refusal would involve no penalty. Adolescents who opted not to participate were allowed to leave the classroom and engage in other school-related activities coordinated by the school administration.

Following the general GSHS study design, the TCI survey was self-administered to students in grades typically attended by adolescents aged 13–17. This corresponded to grades 7–11, known as Forms 1–5 in the TCI. Notably, all students present in the classroom were invited to complete the survey, regardless of age. As measured in the survey, the reported age range spanned from 11 years or younger to 18 years or older, with only a small proportion of students (approximately 3%) falling into the youngest and oldest age categories. Students were nearly equally split across Forms 1–4 (20%−26%). However, many senior (5th-form) students were preparing for external exams and were not in school at the time of the survey. This resulted in lower-than-expected survey participation among this group (8.57%). The student response rate was 66.5%. Thus, the overall survey response rate was 59% (school response rate × student response rate). A total of 1,684 students completed the survey. The survey, including ethical considerations, was approved by the Ministry of Health and Human Services and the Minister of Education, Youth, Sports, and Social Services. Moreover, this study and the use of the secondary TCI GSHS data were approved by the Institutional Review Board of the first author's institution (IRB-2025-708). See Bischops et al. (27) for a description of the GSHS procedures.

### 2.2 Study measures

#### 2.2.1 Demographic variables

The TCI GSHS instrument included two demographic questions: age and sex (male/female). Additionally, in the dietary habits section of the survey, adolescents were asked how often they had gone hungry in the previous 30 days due to insufficient food at home. Response options ranged from 1 “Never” to 5 “Always”. We used this question on food scarcity to create a proxy socioeconomic status (SES) variable with two categories: low SES, defined as “sometimes” to “always” going hungry, and high SES, defined as “rarely” or “never” going hungry (24). A complete list of the survey questions and response options is provided in Table 1.

**Table 1:** Items, response options, and recoding procedure for variables utilized in the study.

Variables	Question	Response options and recoding
Age	How old are you?	A = 11 years or younger, B = 12 years, C = 13 years, D = 14 years, E = 15 years, F = 16 years, G = 17 years, H = 18 years or older. (Recode: A–B = less than 13 years; C–E =13–15 years; F–H = 16 years and older)
Sex	What is your sex?	A = male, B = female
15.5-7.4,-26.5175.3mmFood scarcity	During the past 30 days, how often did you go hungry because there was not enough food in your home?	A = never, B = rarely, C = sometimes, D= most of the time, E = always. (Recode: A–B = no; C–E = yes)
Bullying victimization: during the past 30 days, were you…		
Bullied at school	Bullied on school property?	A = yes, B = no
Bullied out-of-school	Bullied when you were not on school property?	
Cyberbullied	Cyberbullied?	
Psychological distress: during the past 12 months…		
Lonely	How often did you feel lonely?	A = never, B = rarely, C = sometimes, D = most of the time, E = always. (Recode: A–B = no; C–E = yes)
Worried	How often were you so worried about something that you could not sleep at night?	
Consider suicide	Did you seriously consider attempting suicide?	A = yes, B = no
Plan suicide	Did you make a plan about how you would attempt suicide?	
Attempt suicide	How many times did you attempt suicide?	A = 0, B =1, C = 2 or 3, D = 4 or 5, E = 6 or more. (Recode: A = no; B–E = yes)
Peer support		
Close friends	How many close friends do you have?	A = 0, B = 1, C = 2, D = 3 or more (Recode: A = no; B–D = yes)
Parent-adolescent relationships: during the past 30 days, how often did your parents/guardians		
Parental support	Understand your problems and worries?	A = never, B = rarely, C = sometimes, D = most of the time, E = always (Responses summed; parental relationship quality range; 3–15)
Parental involvement	Check to see if your homework was done?	
Parental monitoring	Really know what you were doing with your free time?	
Substance use: during the 30 days, on how many days did you….		
Cigarette use	Smoke cigarettes?	A = 0 days, B = 1 or 2 days, C = 3–5 days, D = 6–9 days, E = 10–19 days, F = 20–29 days, G = All 30 days (Recode: A = no; B–G = yes) (Tobacco use = yes to at lease one—cigarette, other smoked tobacco, smokeless tobacco)
Other smoked tobacco use	Use any form of smoked tobacco products other than cigarettes?	
Smokeless tobacco use	Use any form of smokeless tobacco products?	
Alcohol use	Have at least one drink containing alcohol?	
Marijuana use	Use marijuana?	

#### 2.2.2 Bullying

Three types of bullying victimization were measured in the survey: bullying on school premises, bullying off school premises, and cyberbullying. For the survey, bullying was defined as when someone teases, threatens, ignores, spreads rumors about, calls someone a bad name, makes sexual remarks, or hits, shoves, or hurts another person repeatedly. Physical fighting was not included in the definition, i.e., when two people of about the same strength or power argue, fight, or tease each other in a non-friendly way. Additionally, cyberbullying was defined as a form of bullying using social media or other forms of online communication. In three separate questions, students were asked whether they had experienced each of the three forms of bullying in the past year (yes/no). For the current study, we created a dichotomous variable indicating whether participants experienced at least one form of bullying.

#### 2.2.3 Peer support

We measured peer support with a single question about the number of close friends (0 to 3 or more). We then dichotomized their responses based on whether they had “0” or at least one friend.

#### 2.2.4 Parent-adolescent relationship quality

We measured parent-adolescent relationship quality using three questions that assessed the extent to which parents understood their adolescents' problems and worries (parental support), checked if their homework was completed (parental involvement), and knew what their adolescents were doing with their free time (parental monitoring) (28). The response options for all three items ranged from 1: “never” to 5: “always.” The three items were summed to yield a parental relationship quality score ranging from 3 to 15, with higher scores indicating greater relationship quality (α = 0.64).

#### 2.2.5 Psychological distress

We measured psychological distress using five items (α = 0.73). The first two items assessed the degree to which anxiety disrupted regular sleep and the degree of loneliness. Responses were measured on a five-point Likert scale ranging from 1: “never,” to 5: “always.” A dichotomous variable was created for these two questions, whereby “sometimes,” “most of the time,” and “always” denoted the presence of the mental health attribute, while “rarely” to “never” denoted the absence. The other three items related to whether (yes/no) they considered, planned, or attempted suicide. Psychological distress was considered the presence of at least two of the five mental health indicators (29).

#### 2.2.6 Substance use

We considered the recent use of three substances: alcohol, tobacco, and marijuana. For alcohol use and marijuana use, students were asked how many days they used each substance in the past 30 days, with response options ranging from “0 days” to “all 30 days.” Dichotomous variables were then created for each substance use outcome. Specifically, students who responded “0 days” were classified as having no recent use, and students responding otherwise (i.e., at least 1 day of substance use) were classified as having recent use. For tobacco use, three similar questions were asked about specific tobacco products: cigarettes, smokeless, and all other forms. Recent tobacco use was defined as using at least one form of tobacco in the past 30 days.

### 2.3 Statistical analysis

The GSHS is a nationally representative survey that includes study weights to account for non-responding schools and students, as well as the population distribution by grade and sex. We incorporated these weights, along with a variable indicating adolescents' clustering within schools, into all analyses. We used Stata version 18.0 (StataCorp, College Station, TX, USA) for all statistical analyses. Our analytic approach began with descriptive analyses to understand the prevalence of substance use behaviors and the contextual factors that may influence them. We then conducted bivariate analyses to determine if demographic characteristics and contextual factors differed according to whether the adolescents reported substance use. Specifically, Pearson's chi-square test was used for all categorical variables, and *t*-tests were used for the continuous variable (parental adolescent relationship quality). To account for multiple comparisons across the bivariate analysis, we evaluated statistical significance using a Bonferroni-adjusted threshold (α = 0.05/48 = 0.001).

Finally, logistic regression models were fitted for dichotomous outcomes. Listwise deletion would have excluded 10%−14% of observations, and patterns of missingness suggested that the assumption of missing completely at random (MCAR) was unlikely. Under a missing-at-random (MAR) assumption, multiple imputation by chained equations with 20 imputations was used to address missing values in both predictors and outcomes. All variables included in the final regression models were incorporated into the imputation models. Logistic regression models were then estimated within each imputed dataset, and estimates were pooled across imputations using Rubin's rules (30). Standard errors were calculated using Taylor series linearization to account for survey design. Models included demographic variables, the single-item peer support measure, and the multi-item constructs for psychological distress, bullying victimization, and parent–adolescent relationship quality. Results are reported as odds ratios (ORs) with 95% confidence intervals (CIs). Variance inflation factors (Mean VIF = 1.02; maximum VIF = 2.04; minimum VIF = 1.35) indicated that multicollinearity was not a concern. Among the variables included in the logistic models, the strongest correlation was found between the bully victimization and psychological distress domains (*r* = 0.28, *p* < 0.001). Supplementary Table 1 provides the full correlation matrix.

## 3 Results

### 3.1 Characteristics of study participants

Table 2 presents the distributions of demographic, socioecological, and substance-use variables. A nearly equal proportion of adolescents identified as male (49.82%) and female (50.18%). Additionally, most students were between 13 and 15 years old (68.55%), and 35.19% reported low SES (i.e., “sometimes” to “always” going hungry because there was no food at home).

**Table 2:** Demographic, socio-ecological factors, and substance use prevalence of adolescents who completed the TCI global school-based health survey in 2022.

Domain	Variable	Values	Percentage^a^	95% CI
Demographics	Sex	Female	50.18	46.71–53.65
		Male	49.82	46.35–53.29
	Age group	≤ 12 years	12.15	08.39–17.26
		13–15 years	68.55	63.11–73.52
		≥16 years	19.31	15.25–24.14
	Food scarcity	No	64.81	61.46–58.02
		Yes	35.19	31.98–38.54
Mental health indicators	Loneliness	No	67.33	64.28–70.24
		Yes	32.67	29.76–35.72
	Worried	No	77.68	74.88–80.26
		Yes	22.32	19.74–25.12
	Considered suicide	No	71.33	68.39–74.09
		Yes	28.67	25.91–31.61
	Planned suicide	No	73.17	70.11–76.03
		Yes	26.83	23.97–29.89
	Attempted suicide	No	77.69	75.18–80.01
		Yes	22.31	19.99–24.82
	Psychological distress	No	65.21	61.88–68.39
		Yes	34.79	31.61–38.12
Bully victimization	Bullied at school	No	75.65	71.77–78.31
		Yes	24.35	21.69–27.23
	Bullied out-of-school	No	84.45	82.19–86.47
		Yes	15.55	13.53–17.81
	Cyberbullied	No	83.45	81.17–85.50
		Yes	16.55	14.50–18.83
	Any bully victimization	No	63.94	60.87–66.91
		Yes	36.06	33.09–39.13
Peer support	*Close friends*	None	8.21	6.53–10.27
		At least 1	91.79	89.73–93.47
Parental—adolescent relationship characteristics	Parental support [mean (SE)]		2.38 (0.04)	2.30–2.46
	Parental involvement [mean (SE)]		2.76 (0.04)	2.68–2.84
	Parental monitoring [mean (SE)]		3.03 (0.04)	2.95–3.11
	PARQ [mean (SE)]		7.95 (0.10)	7.76–8.14
Substance use	Alcohol	No	70.89	67.69–73.91
		Yes	29.11	26.09–32.31
	Tobacco	No	87.95	85.91–89.73
		Yes	12.05	10.27–14.09
	Marijuana	No	90.99	88.66–92.87
		Yes	9.01	7.13–11.34

^a^Represents weighted proportions.

PARQ, parent adolescent relationship quality.

In terms of the mental health indicators, 32.67% of respondents reported being lonely, while 22.32% reported being so worried that it led to insomnia. Suicidal ideation and behaviors, that is, considering and planning, and attempting, were reported by 28.67, 26.83, and 22.31% of respondents, respectively. About a third of adolescents (34.79%) reported at least two of these mental health indicators (psychological distress). Concerning bully victimization, students were more likely to be bullied on school premises (24.35%) compared to off-school premises (15.55%) and via online avenues (cyberbullied; 16.55%). Thirty-six percent (36.06%) reported at least one type of bullying victimization. A small proportion (8.21%) reported having no close friends, and the mean parental relationship quality score was 7.95 (SE = 0.10). Finally, alcohol, tobacco, and marijuana use were reported by 29.11, 12.05, and 9.01% of respondents, respectively.

### 3.2 Bivariate analyses

Bivariate results are reported in Table 3.

**Table 3:** Prevalence of substance use (alcohol, tobacco, and marijuana) by socioecological factors among adolescents who completed the TCI global school-based health survey in 2022.

Variables	Category	Alcohol use^a^		Tobacco use^a^		Marijuana use^a^	
		%	χ^2^ or *t* (*p*)	%	χ^2^ or *t* (*p*)	%	χ^2^ or *t* (*p*)
Demographics							
Age group (years)	≤ 12	19.00	27.97 (< 0.001)	11.15	3.55 (0.30)	3.29	25.65 (< 0.001)
	13–15	26.92		11.57		8.00	
	≥16 years	39.73		15.38		15.75	
Sex	Female	30.89	3.43 (0.11)	11.55	0.10 (0.10)	8.69	0.25 (0.53)
	Male	26.65		12.06		9.42	
Food scarcity	No	28.84	0.14 (0.69)	11.28	1.76 (0.22)	8.02	3.37 (0.10)
	Yes	29.75		13.50		10.79	
Mental health indicators							
Loneliness	No	26.97	6.77 (0.01)	10.23	9.12 (0.01)	6.25	27.60 (< 0.001)
	Yes	33.35		15.37		14.26	
Worried	No	26.03	24.93 (< 0.001)	10.78	5.68 (0.05)	7.27	17.63 (0.002)
	Yes	39.81		15.31		14.48	
Considered suicide	No	24.10	35.49 (< 0.001)	8.92	18.60 (< 0.001)	6.57	15.85 (< 0.001)
	Yes	39.24		16.52		12.74	
Planned suicide	No	24.43	35.86 (< 0.001)	8.32	26.13 (< 0.001)	6.53	22.41 (< 0.001)
	Yes	40.05		17.16		17.11	
Attempted suicide	No	25.66	30.34 (< 0.001)	9.08	39.04 (< 0.001)	6.13	37.38 (< 0.001)
	Yes	40.90		20.95		13.55	
Psychological distress^b^	No	23.92	39.15 (< 0.001)	8.95	28.57 (< 0.001)	5.80	39.24 (< 0.001)
	Yes	38.94		17.87		15.25	
Bullying victimization							
Bullied at school	No	27.90	1.08 (0.37)	9.77	5.19 (0.05)	8.82	1.04 (0.32)
	Yes	30.69		13.87		7.15	
Bullied out of school	No	27.43	5.84 (0.02)	9.87	18.72 (< 0.001)	8.63	0.02 (0.90)
	Yes	35.17		19.25		8.92	
Cyber-bullied	No	28.10	0.73 (0.40)	10.15	6.30 (0.02)	8.48	0.09 (0.78)
	Yes	30.79		15.41		7.93	
Any bully victimization	No	27.00	3.50 (0.13)	9.57	6.39 (0.05)	9.22	2.45 (0.16)
	Yes	31.47		13.63		6.94	
Peer support							
Close friend(s)	No	29.79	0.09 (0.82)	13.78	0.65 (0.40)	16.72	11.15 (0.01)
	Yes	28.52		11.45		8.01	
Parent-adolescent relationship quality							
Parental support^b^		2.14	4.02 (< 0.001)	1.87	6.57 (< 0.001)	1.80	5.27 (< 0.001)
Parental involvement^b^		2.60	2.41 (0.02)	2.49	2.36 (0.04)	2.28	4.34 (< 0.001)
Parental monitoring^b^		2.75	4.44 (< 0.001)	2.47	5.12 (< 0.001)	2.46	4.32 (< 0.001)
PARQ^b^		7.28	4.64 (< 0.001)	6.42	6.98 (< 0.001)	6.29	7.69 (< 0.001)

^a^Represents weighted proportions.

^b^Mean values provided instead of proportions.

PARQ, parent adolescent relationship quality.

#### 3.2.1 Alcohol use

Neither sex nor food scarcity was associated with alcohol use. However, an association with age group was observed (χ^2^ = 27.97, *p* < 0.001); alcohol consumption was highest among adolescents aged 16 years and older (39.73%). All mental health indicators, including whether they were determined to have psychological distress, were associated with alcohol use (*p* < 0.001). The only exception was loneliness. None of the bullying victimization indicators were associated with alcohol use. Neither was the single-item measure of peer support. On the other hand, adolescents who reported alcohol use had significantly lower mean parental support (*t* = 4.02, *p* < 0.001), a lower parental monitoring score (*t* = 4.44, *p* < 0.001), and a lower overall parental-adolescent relationship quality score (*t* = 4.64, *p* < 0.001).

#### 3.2.2 Tobacco use

Tobacco use did not differ significantly by sex, age, or SES. However, it was associated with three of the five mental health indicators: considering suicide (χ^2^ = 18.60, *p* < 0.001), planning suicide (χ^2^ = 26.13, *p* < 0.001), and attempting suicide (χ^2^ = 39.04, *p* < 0.001). It was also associated with the assessment of psychological distress (χ^2^ = 28.57, *p* < 0.001). Among the bullying victimization indicators, only being bullied off school premises was associated with tobacco use (χ^2^ = 18.72, *p* < 0.001). Tobacco use was also not associated with parental involvement, although it was associated with lower mean parental support (*t* = 6.57, *p* < 0.001), lower parental monitoring (*t* = 5.12, *p* < 0.001) scores, and a lower overall parental relationship quality score (*t* = 6.98, *p* < 0.001).

#### 3.2.3 Marijuana use

Marijuana use was associated with older age (χ^2^ = 25.65, *p* < 0.001) but not sex or food scarcity. It was also associated with four of the five mental health indicators: loneliness (χ^2^ = 27.60, *p* < 0.001), considering suicide (χ^2^ = 15.85, *p* < 0.001), planning suicide (χ^2^ = 22.41, *p* < 0.001), and attempting suicide (χ^2^= 37.38, *p* < 0.001) and with the overall assessment of psychological distress (χ^2^ = 39.24, *p* < 0.001). Marijuana use was not associated with any of the bullying victimization indicators or peer support. However, it was also associated with all parental- adolescent relationship quality indicators; parental support (*t* = 5.27, *p* < 0.001), involvement (*t* = 4.34, *p* < 0.001), and monitoring (*t* = 4.32, *p* < 0.001) scores and with an overall lower parental relationship quality score (*t* = 7.69, *p* < 0.001).

### 3.3 Multivariable analyses

The results of multivariable analysis are presented in Table 4. When other factors in the model were controlled for, alcohol use was associated with age, psychological distress, and parent adolescent relationship quality. Adolescents between the ages of 13 and 15 years (aOR = 1.66, 95% CI = 1.13–2.45) and 16 years and older (aOR = 2.46, 95% CI = 1.58–3.86) had significantly higher odds of consuming alcohol than younger adolescents. Moreover, adolescents with psychological distress had higher odds of alcohol use than those who were determined not to have psychological distress (aOR = 1.64, 95% CI = 1.28–2.11). Finally, as the parent-adolescent relationship quality score increased, the odds of alcohol use decreased (OR = 0.93, 95% CI = 0.90–0.97).

**Table 4:** Odds ratios and 95% confidence intervals resulting from logistic regression analysis of demographic and socioecological predictors of substance use.

Variable	Category	Alcohol		Tobacco		Cannabis	
		aOR	95% CI	aOR	95% CI	aOR	95% CI
Sex	Female	—	—	—	—	—	—
	Male	0.93	0.74–1.17	1.19	0.86–1.64	1.50^*^	1.02–2.21
Age group	≤ 12 years	—	—	—	—	—	—
	13–15 years	1.66^*^	1.13–2.45	1.08	0.65–1.80	2.19	0.93–5.15
	≥16 years	2.46^***^	1.58–3.86	1.22	0.68–2.18	3.82^*^	1.54–9.44
Food scarcity	No	—	—	—	—	—	—
	Yes	0.91	0.71–1.15	0.99	0.72–1.36	1.08	0.73–1.59
Bully victimization	No	—	—	—	—	—	—
	Yes	1.14	0.90–1.45	1.24	0.88–1.75	0.53^**^	0.34–0.83
Psychological distress	No	—	—	—	—	—	—
	Yes	1.64^***^	1.28–2.11	1.78^**^	1.27–2.50	3.14^***^	2.10–4.68
Peer support	No	—	—	—	—	—	—
	Yes	1.34	0.88–2.05	0.97	0.57–1.66	0.68	0.38–1.20
PARQ		0.93^**^	0.90–0.97	0.88^***^	0.83–0.92	0.86^***^	0.80–0.91

^*^p < 0.05, ^**^p < 0.01, ^***^p < 0.001.

PARQ, parent adolescent relationship quality.

In the tobacco model, only psychological distress and parental involvement were associated with use. Psychological distress (aOR = 1.78, 95% CI = 1.27–2.50) was associated with an increased odds of use, whereas a higher parental relationship quality score (aOR = 0.88, 95% CI = 0.83–0.92) was associated with a lower odds of use. Finally, in the marijuana model, sex, age, bullying victimization, psychological distress and parental relationship quality were associated with use. Males (aOR = 1.50, 95% CI = 1.02–2.21) and adolescents 16 years and older (aOR = 3.82, 95% CI = 1.54–9.44) had higher odds of use. Additionally, adolescents reporting bullying victimization had lower odds of marijuana use (OR = 0.53, 95% CI = 0.34–0.83). Finally, psychological distress (aOR = 3.14, 95% CI = 2.10–4.68) was associated with a higher odds of use, and higher parental involvement scores (aOR = 0.86, 95% CI = 0.80–0.91) were associated with a lower odds of marijuana use.

## 4 Discussion

As hypothesized, TCI adolescent substance use prevalence rates were closer to regional averages than global averages. The 30-day alcohol use prevalence (29.11%) was slightly below the regional average (34.9%) but surpassed the global average of 25% (4). Regionally, it was closest to rates reported by the British Virgin Islands (29.7%), another British Overseas Territory with a similar population size, and the Bahamas (27%), the geographically closest country with similar cultures and values. Though less comprehensive data are available at the country level for the other substances, recent tobacco (12.11%) and marijuana use (9.01%) surpassed both the regional (11.3 and 6.6%, respectively) and global averages (10.3 and 4.3%, respectively) (5, 6). However, caution must be exercised in such comparisons, as the regional and global rates are based on data collected well before 2022 (2006–2018). Our findings are notable because they are based on the only nationally representative survey of health risk behaviors, and the responses reflect input from over half of the country's adolescents. Thus, they provide the first robust examination of the prevalence of adolescent substance use in TCI. They suggest that substance use among TCI adolescents is a public health problem that requires attention.

We found that older adolescents are more likely to report alcohol and marijuana use than younger adolescents. This is consistent with our hypothesis and with observations among adolescents in Jamaica, but not in Barbados (18, 20, 21). Regional differences may reflect cultural contexts. For instance, older TCI adolescents may adhere to the traditional pattern in which they are more strongly influenced by peers than by parents, experience greater autonomy, and report increased access to substances (31, 32). However, across the region, parents exert varying levels of control over older adolescents. For instance, Lipps et al. (33) found that authoritative parenting was most reported in the Bahamas, Jamaica, and St. Kitts and Nevis, whereas neglectful parenting was most prevalent in St. Vincent. There are also varying levels of enforcement of age-related substance use restrictions, which may help to explain regional differences in associations between adolescent age and substance use (34).

Our null findings in relation to sex and alcohol and tobacco use are consistent with what was found in Jamaica (20). Historically, boys have reported higher levels of risk behaviors than girls, a pattern often attributed to gendered socialization processes in which girls were subject to greater social control, while boys were typically afforded more autonomy and freedom to behave with fewer restrictions (35). Walcott et al. (36) found that high support for masculinity and inequitable gender norms was associated with engagement in risky behavior among males in Jamaica. In recent decades, however, gender convergence in substance use has been linked to greater behavioral freedom for girls (35). Although progress has not been uniform across the region, LAC has made substantial advances in gender equality, including increased enrollment in formal education, greater labor force participation, and improved representation in public leadership roles. Significant efforts have also been made to strengthen women's voice and agency, with a focus on preventing gender-based violence and addressing harmful norms of masculinity (37). We did find an association between sex and marijuana use, which is contrary to studies in Jamaica and Barbados (18, 20). Differences in sex-substance use associations across the region may reflect differing patterns of female autonomy. In the TCI, the gender gap may be narrowing, though more slowly for substances perceived as higher risk. In some contexts, marijuana is viewed as posing greater physical and mental risks compared to alcohol and tobacco (38, 39).

Contrary to our hypothesis and what was found in St. Vincent, our study did not find an association between bullying victimization and alcohol or tobacco use (22). These country-level differences may reflect cultural variations in how bullying is perceived and addressed. Adolescents in the TCI may exemplify the perspective proposed by Arcadepani et al. (40), in which positive cultural and family values foster resilience and help buffer the negative effects of bullying involvement. Unexpectedly, we found a reverse association between bullying victimization and marijuana use. Although inconsistent with studies in the region (21), Priesman et al. (41) reported a similar finding using data from the United States Youth Risk Behavior Survey (YRBS). They suggest that those who are traditionally bullied have reduced access to marijuana through their peer groups, as they tend to be less socially connected. Consistent with this, most adolescent marijuana users in the Caribbean obtain marijuana by sharing or receiving it from friends (42). Beyond its impact on access, social isolation itself, particularly due to peer exclusion, has been associated with lower substance use and may independently contribute to this relationship (43). The observed relationship may also reflect measurement limitations and residual confounding.

The positive association between psychological distress and substance use has been more consistently observed across the region (44). One explanation is that adolescents with poorer mental health may be more likely to engage in substance use as a coping strategy to alleviate tension or escape negative emotions (45). However, given the cross-sectional design of this study, reverse causality is also plausible; substance use may contribute to or exacerbate mental health symptoms (46). This relationship may also reflect shared underlying vulnerabilities, as both psychological distress and substance use are often linked to factors such as stress, trauma, and adverse childhood experiences (47).

We also found that close parental relationships were protective against all forms of substance use. Although this finding supports our hypothesis, similar associations have not been consistently reported across the region. Ruprah et al. (23) found that positive parental relationships protect against substance use in most but not all the Caribbean countries, even when consistently measured, suggesting that the link is context-driven. Differences may specifically lie in the nature of the relationship and parenting practices, which vary across countries (33). In certain settings, potentially including TCI, high-quality family relationships characterized by emotional warmth, affection, and open and supportive communication may mitigate adolescent stress by facilitating adaptive coping, providing emotional support through active listening, and protecting against depressive symptomatology (48). Each of these factors may lead to reduced substance use. Unlike other studies in the region and contrary to our hypothesis, we did not find a link between peer support and substance use (18, 21). This could be related to how peer support was measured across countries—the quantity of friends may not be as relevant as the types of friends. For instance, Griffith and Jackman (18) found that adolescents with marijuana-using friends in Barbados were more likely to report marijuana use. In contrast, those with peers who disapprove of marijuana use were less likely to report marijuana use.

### 4.1 Limitations

Our findings should be considered in light of the study's limitations. First, the overall student participation rate (59%) was low. However, this limitation might have been mitigated by the census approach combined with study weights. We also employed a cross-sectional design, which made it difficult to determine the temporal nature of the relationships. Furthermore, the standardized questionnaire enabled cross-country/region comparisons but did not allow the development of specific questions based on our hypotheses. For example, in line with the GSHS core questionnaire, information on race/ethnicity was not collected, limiting our ability to examine how substance use prevalence and its correlates may vary across racial or ethnic groups. It is worth noting that the overall racial and ethnic profile of the TCI comprises Black (87.6%), White (7.9%), mixed (2.5%), East Indian (1.3%), and other (0.7%) (49).

Measurement limitations should also be considered. Psychological distress was assessed using a composite measure that combines heterogeneous constructs (e.g., loneliness and suicidality), which may not fully capture the multidimensional nature of mental health and could introduce misclassification. Socioeconomic status was approximated using food scarcity, an imperfect proxy that may not reflect broader material or social resources. Peer support was measured using a single-item indicator of the number of friends, which may not adequately capture the quality or functional aspects of social relationships. Additionally, the internal consistency of the parental relationship quality scale was modest (α = 0.65); however, Cronbach's alpha is sensitive to scale length and may underestimate reliability for brief measures. Collectively, these limitations may introduce measurement error, bias associations toward the null, and reduce comparability with studies using more comprehensive measures. As such, findings should be interpreted with appropriate caution. Finally, the study was limited to high school students and excluded adolescents who were not enrolled in high school. However, like many countries, TCI has compulsory school attendance for school-age children and employs truancy officers to enforce compliance. Thus, less than 1% of adolescents and youth were reported to be out of school in 2020 (50).

### 4.2 Strengths and implications

Despite its limitations, this study has several notable strengths. It is the first to estimate the prevalence and correlates of substance use among a nationally representative sample of adolescents in the TCI and among the earliest studies in Latin America and the Caribbean (LAC) conducted shortly after the peak of the COVID-19 pandemic. Latin America and the Caribbean (LAC) accounted for approximately 15% of global COVID-19 cases and 28% of deaths, despite representing only about 8%−9% of the world's population—highlighting a disproportionate burden of disease in the region (51). Globally, children and adolescents have been particularly vulnerable to the indirect effects of the pandemic, including disruptions to education systems, increases in anxiety, depression, and loneliness, financial instability, and increased exposure to family stress and violence; all of which may impact adolescent substance use patterns (52–54). Adolescents in LAC, particularly in small island developing states, are especially sensitive to such shocks due to pre-existing vulnerabilities, including recurrent exposure to natural disasters, constrained health system capacity, and economic dependence on tourism (55, 56). Understanding substance use patterns in the post-pandemic context is therefore critical.

Given that substance use is a a major cause of preventable death and a significant contributor to the global burden of disease, the above global prevalence rates we observed underscore the need for a national response (57). In the TCI context, psychological distress and the quality of parental relationships emerged as consistent correlates across substances, suggesting these factors should be prioritized in prevention efforts. We recommend implementing standardized screening tools to identify high-risk adolescents and facilitate referral to health professionals for individualized prevention and treatment services. The WHO/PAHO *No Time to Lose* report (58) highlights the importance of early interventions, including school-based mental health services, to prevent adolescents from using substances as a coping mechanism for emotional and psychological challenges.

Additionally, family-based interventions, which have been successfully designed and evaluated in diverse settings, may help strengthen family bonds, fostering environments where adolescents feel safe, secure, and supported—thereby reducing vulnerability to substance use, especially in contexts with strong external social pressures. Since both male and female adolescents in the TCI report high substance use rates, interventions should target all genders, with older adolescents being a particular focus for alcohol reduction strategies. These findings may also provide useful guidance for other small territories in the region.

## 5 Conclusions

The WHO (10) emphasizes country-level responsibility for formulating, implementing, monitoring, and evaluating public policies to reduce harmful substance use to achieve SDG 3.5 (10). Recommended policy strategies include regulating the marketing of substances to youngsters, restricting their availability, and reducing demand through taxation and pricing mechanisms. Programmatic approaches may include implementing screening and brief intervention programs. Our study found links between substance use and several socio-ecological factors, which can offer insights into the most effective approaches. Links with demographic factors provide opportunities to target at-risk youth, while links with other sociological factors demonstrate pathways for action. Future studies should explore deeper cultural factors and social experiences that influence substance use to ensure the interventions are targeted and culturally sensitive. Finally, we recommend that future iterations of the TCI GSHS examine trends over time and longitudinal studies to establish temporal relationships.

## Data Availability

The datasets presented in this article are not readily available consistent with GSHS procedures, the Turks and Caicos Islands retains exclusive access to the data for an embargo period before public release. The data will be publicly available in 2026 on the WHO website: https://www.who.int/teams/noncommunicablediseases/surveillance/data. Requests for access to this dataset before it becomes publicly available should be directed to the Ministry of Health and Human Services in the Turks and Caicos Islands, MOHAHS@gov.tc.
