# Supplementary material for: Prevalence and socio-ecological correlates of adolescent alcohol, tobacco, and marijuana use in the Turks and Caicos Islands
Source: Front Public Health. 2026 May 29;14:1747234. doi: 10.3389/fpubh.2026.1747234 (PMC13259790; doi:10.3389/fpubh.2026.1747234)
Supplement: Supplementary file 1 [file Table_1.docx]

**Supplemental Table 1**: Correlation matrix including demographic and contextual variables entered into the logistic regression model. TCI Global School Health Survey 2022

|  | Sex | Age group | Peer support | Food Scarcity | Parent-Adolescent Relationship Quality | Psychological Distress | Any Bullying Victimization |
| --- | --- | --- | --- | --- | --- | --- | --- |
| Sex | 1 |  |  |  |  |  |  |
| Age Group | 0.0126 | 1 |  |  |  |  |  |
| Peer Support | -0.0123 | -0.0755** | 1 |  |  |  |  |
| Food Scarcity | 0.0017 | 0.0096 | -0.0543* | 1 |  |  |  |
| Parent-Adolescent Relationship Quality | 0.0804* | -0.1267*** | 0.0691* | -0.1441** | 1 |  |  |
| Psychological Distress | -0.2535*** | 0.0686* | -0.0429 | 0.1434*** | -0.2715*** | 1 |  |
| Any Bullying Victimization | -0.122*** | -0.0483* | -0.0963** | 0.0705** | -0.1145*** | 0.2782*** | 1 |

**Note.** Values represent weighted Pearson correlation coefficients accounting for the complex survey design

*p < .05, **p < .01, *** p < .001
